# P-53. Analyzing the Risks of Complicated S.pneumoniae Bacteremia: Community Vaccine Uptake and Preventive Measures in Real-World Scenarios

**DOI:** 10.1093/ofid/ofaf695.282

**Published:** 2026-01-11

**Authors:** Rashini Jayawardena, Christian Mark Gill, Robin R Chamberland, Nongnooch Poowanawittayakom

**Affiliations:** Saint Louis University School of Medicine, st.louis, MO; SSM Health Saint Louis University Hospital, St. Louis, Missouri; Saint Louis University School of Medicine, st.louis, MO; Washington University , st.louis, MO

## Abstract

**Background:**

Invasive pneumococcal bacteremia may manifest in approximately 20-25% of pneumococcal patients, leading to a mortality rate of about 20-30%. This retrospective study examines the factors contributing to uncomplicated versus complicated pneumococcal bacteremia, with a focus on cardiac involvement and the role of vaccines in prevention within communities.

**Figure:**
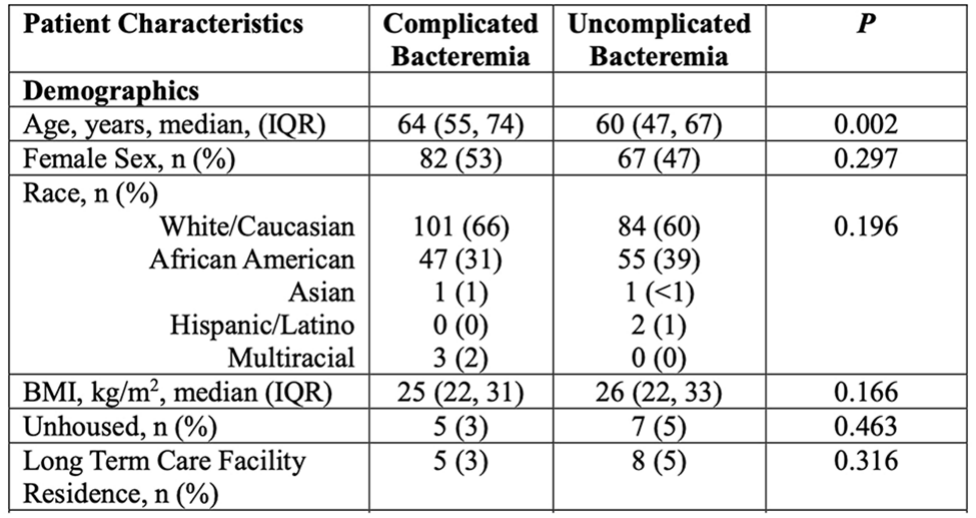
Patient Characteristics

Vaccines status

**Figure:**
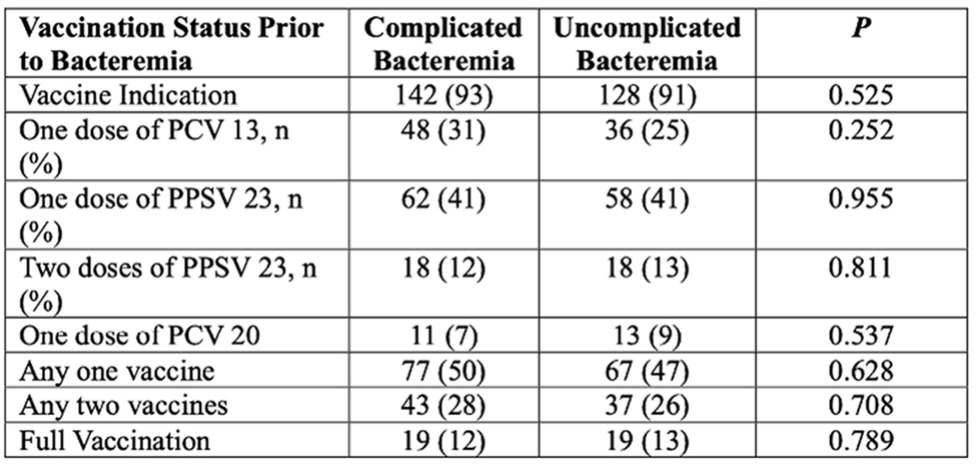

**Methods:**

A 6-year retrospective review of data was collected from the medical records of patients admitted to SSM and Saint Louis University between 2018 and 2024. Complicated bacteremia was defined as any occurrence in which bacteremia leads to secondary complications or involves other parts of the body, such as the heart, brain, bones, or other organs. SPSS was utilized to perform statistical analysis on the variables.

**Results:**

Patients with a median age of 64 years (IQR 19) were found to have a higher incidence of complicated bacteremia (P = 0.002). Those with a median height of 167.6 cm (IQR 16.6) and a median weight of 72.6 kg (IQR 28.4) also showed significant relationships, with p-values of 0.005 and 0.009, respectively. The following conditions were found to have statistically significant relationships with complicated bacteremia: septic arthritis 3.9% (6/153) (P = 0.017), septic emboli 3.9% (6/153) (P = 0.017), endocarditis 9.8% (15/153) (P < 0.001), empyema 94.1% (144/153) (P < 0.001), and pneumonia 90.8% (139/153) (P < 0.001). Additionally, a median albumin level of 3 (IQR 0.9) (P = 0.005), a median WBC count of 15.1 (IQR 13.8) (P = 0.005), and ICU admission 54.9% (84/153) (P = 0.002) were all significantly associated with complicated bacteremia.

In the present study, vaccination was not associated with less complicated BSI. Several factors may account for this observation and require further investigation. Despite this, only 12% and 13% of patients received vaccines consistent with full vaccination. Approximately half of each group received at least one pneumococcal vaccine.

**Conclusion:**

The data suggest that older age may be associated with a higher incidence of complicated bacteremia.Vaccine promotions and further study needs to evaluate the real-world data of vaccines efficacy to prevent complicated S.pneumoniae bacteremia.

**Disclosures:**

Christian Mark Gill, PharmD, BCIDP, Cepheid: Grant/Research Support|Cumberland Pharmaceuticals: Grant/Research Support|Entasis: Grant/Research Support|Everest Medicines: Grant/Research Support|Shionogi: Grant/Research Support Robin R. Chamberland, PhD D(ABMM), bioMerieux: Advisor/Consultant|Pattern Bioscience, Inc.: Advisor/Consultant|Pattern Bioscience, Inc.: Grant/Research Support

